# Interphotoreceptor Retinoid-Binding Protein (IRBP) in Retinal Health and Disease

**DOI:** 10.3389/fncel.2020.577935

**Published:** 2020-11-19

**Authors:** Shaoxue Zeng, Ting Zhang, Michele C. Madigan, Nilisha Fernando, Riemke Aggio-Bruce, Fanfan Zhou, Matthew Pierce, Yingying Chen, Lianlin Huang, Riccardo Natoli, Mark C. Gillies, Ling Zhu

**Affiliations:** ^1^Save Sight Institute, The University of Sydney, Sydney, NSW, Australia; ^2^Department of Ophthalmology, West China Hospital, Sichuan University, Chengdu, China; ^3^School of Optometry and Vision Sciences, University of New South Wales, Sydney, NSW, Australia; ^4^The John Curtin School of Medical Research, The Australian National University, Acton, ACT, Australia; ^5^The Australian National University Medical School, The Australian National University, Acton, ACT, Australia; ^6^Sydney Pharmacy School, The University of Sydney, Sydney, NSW, Australia

**Keywords:** IRBP gene, photoreceptor degeneration, visual cycle, gene therapy, retinoid

## Abstract

Interphotoreceptor retinoid-binding protein (IRBP), also known as retinol binding protein 3 (RBP3), is a lipophilic glycoprotein specifically secreted by photoreceptors. Enriched in the interphotoreceptor matrix (IPM) and recycled by the retinal pigment epithelium (RPE), IRBP is essential for the vision of all vertebrates as it facilitates the transfer of retinoids in the visual cycle. It also helps to transport lipids between the RPE and photoreceptors. The thiol-dependent antioxidant activity of IRBP maintains the delicate redox balance in the normal retina. Thus, its dysfunction is suspected to play a role in many retinal diseases. We have reviewed here the latest research on IRBP in both retinal health and disease, including the function and regulation of IRBP under retinal stress in both animal models and the human retina. We have also explored the therapeutic potential of targeting IRBP in retinal diseases. Although some technical barriers remain, it is possible that manipulating the expression of IRBP in the retina will rescue or prevent photoreceptor degeneration in many retinal diseases.

## Introduction

### A Brief History of IRBP

Interphotoreceptor retinoid-binding protein (IRBP), also known as retinol binding protein 3 (RBP3), was first discovered in soluble proteins extracted from the bovine interphotoreceptor matrix (IPM), which is located between the neural retina and the retinal pigment epithelium (RPE) (Adler and Severin, 1981). Several unknown proteins were identified, including a 140 kilodalton (kDa) protein (Liou et al., 1982). Liou et al. suggested that this 140 kDa protein might be a transporter protein transferring retinol between the outer segments of rod photoreceptors and the RPE (Liou et al., 1982). They postulated that the molecular weight of IRBP is 260 kDa in its glycosylated form, while its backbone is 140–145 kDa (Liou et al., 1982).

In the 1990's, several studies implicated IRBP in retinal development (Gonzalez-Fernandez et al., 1993; Timmers et al., 1993; Liou et al., 1994; Stenkamp et al., 1998). The messenger RNA (mRNA) expression of IRBP in the retina of embryonic mice was low. mRNA transcripts in mice were first detected at embryonic day 11 and continued to increase to its peak expression on postnatal day 4, after which there was a slow decrease and reached constancy by postnatal day 20 (Liou et al., 1994). The protein expression of IRBP increased together with its mRNA level during photoreceptor development in bovine and zebrafish embryos (Timmers et al., 1993; Stenkamp et al., 1998). Protein and mRNA expression were also markedly increased in Sprague Dawley rats between postnatal day 1 and 9, a period critical for outer segment formation. Then, IRBP mRNA levels decreased a little and stabilized by postnatal day 20 when the outer segments achieved their adult length (Gonzalez-Fernandez et al., 1993).

IRBP has been shown to be downregulated in animal models of retinal disease, including Abyssinian cats that carry a homozygous IRBP mutation that causes pan-retinal degeneration (Narfstrom et al., 1989). IRBP downregulation has also been reported in mice with induced Müller cell dysfunction and a streptozotocin (STZ)-induced diabetic rat model (Zhu et al., 2015; Malechka et al., 2017). IRBP deficiency also impaired eye growth and compromised retinal health in mice (Wisard et al., 2011). These reports suggest a correlation between IRBP dysregulation and photoreceptor degeneration.

### Retinal Location of IRBP

**Figure 1:**
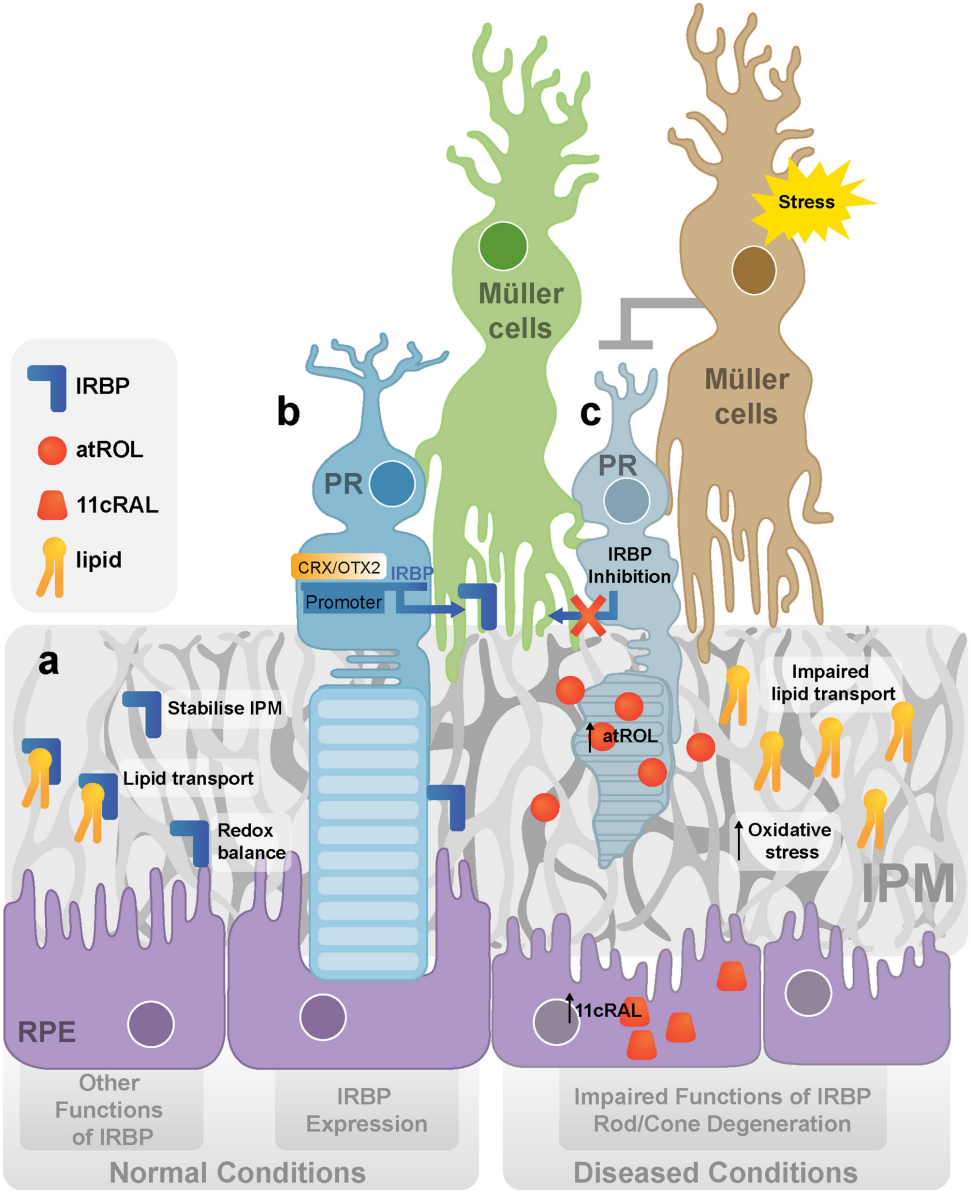
IRBP in normal and diseased retina. **(a)** The expression and functions of IRBP. IRBP is produced and secreted by photoreceptors. The main function of IRBP is to stabilize IPM, transport lipid between photoreceptors and the RPE and maintain the redox balance in the outer retina. **(b)** The regulation of IRBP. OTX2/CRX activates the expression of IRBP by binding to its promoter. **(c)** IRBP dysregulation and rod/cone degeneration under stress. It is proposed that stress activates Müller cells inhibiting the production of IRBP in photoreceptors, leading to the dysfunction of lipid transport, increased oxidative stress, and accumulation of retinoids (atROL). (IRBP, Interphotoreceptor retinoid-binding protein; CRX, cone-rod-homeobox; OTX2, orthodenticle homolog 2; IPM, interphotoreceptor matrix; RPE, retinal pigment epithelium; PR, photoreceptor; atROL, all-*trans*-retinol; 11cRAL, 11-*cis*-retinal).

IRBP is secreted by photoreceptors and accumulates in the IPM to facilitate the transport of material for visual pigment regeneration (Ishikawa et al., 2015) (Figure 1a). It has only been found in vertebrates (Kusakabe et al., 2009). In the eyes of non-vertebrates, an alternative mechanism of pigment regeneration is confined to photoreceptors (Kusakabe et al., 2009). Vertebrates acquired IRBP in the evolution of the visual cycle to accommodate a complicated visual cycle. IRBP can traffick retinoid, a class of Vitamin A derivatives that includes retinol and retinal, between photoreceptors and RPE cells (Kusakabe et al., 2009). IRBP mRNA was detected in both cones and rods of adult Xenopus retina. However, IRBP in the embryo is synthesized by the central retina and diffuses through the matrix, reaching the peripheral retina (Hessler et al., 1996).

Theo and colleagues found IRBP in bovine pineal gland cells by *in situ* hybridization using IRBP cDNA probes. They discovered that IRBP is highly expressed in a population of pineal cells on mRNA level, but they did not quantify its protein expression (van Veen et al., 1986). Expression of IRBP (Rodrigues et al., 1986), cone arrestin (Craft et al., 1994) and opsins (Blackshaw and Snyder, 1997), all photoreceptors markers, were also found in the pineal gland of Rhesus monkeys, rats and catfish. It remains unclear why there is a morphological and possibly functional analogy between photoreceptors and pinealocytes. The pineal gland, a small neuroendocrine organ that synthesizes and secretes melatonin, is also photosensitive in lower vertebrates (Sapède and Cau, 2013). Some researchers have hypothesized that mammalian pinealocytes might have evolved from photoreceptors (Sapède and Cau, 2013).

### Molecular Characteristics and Regulation of IRBP

Mammals have four protein subunits of IRBP (Gonzalez-Fernandez, 2012), each of which consists of ~300 amino acids (Nickerson et al., 2006). Two of the four subunits are similar but have different affinities to all-trans-retinol. Notably, disrupting one subunit does not affect the overall function because other subunits compensate for the dysfunctional subunit (Gonzalez-Fernandez and Ghosh, 2008).

The *IRBP* gene is regulated by cone-rod-homeobox (CRX) and orthodenticle homolog 2 (OTX2), two essential transcriptional factors in photoreceptors (Fei et al., 1999; Nishida et al., 2003) (Figure 1b). Studies have suggested that the “cone-rod-homeobox element” is essential for the photoreceptor-specific activity of the *IRBP* promoter *in vivo*. This element is the major binding site of the CRX, which can directly regulate *IRBP* expression (Fei et al., 1999). OTX2 is an upstream regulator of CRX. Both OTX2 and CRX mRNAs have been identified in adult human retinas (Bobola et al., 1999; Nishida et al., 2003; Li et al., 2015). Overexpressing OTX2 increased *irbp* promoter-luciferase activity by 5–7-fold in WERI-Rb1 retinoblastoma cells, suggesting that OTX2 activates the *irbp* promoter (Bobola et al., 1999). Overall, CRX and OTX2 are both specific gene regulators of IRBP that are critical in photoreceptor development.

## IRBP in the Normal Retina

### Retinoids Transport

Retinoid recycling and metabolism within the eye have been extensively studied for decades. George Wald was awarded a Nobel prize in 1967 for discovering the photoreceptive proteins in the eye, the “chromoproteins” (Wald, 1935). Vision is initiated and maintained by their photolysis and regeneration in the visual cycle (Kiser et al., 2014). Photolysis of 11-*cis*-retinal is the only reaction that converts light signals into electrical signals in vertebrate photoreceptors (Molday and Moritz, 2015). Circulation and regeneration of 11-cis-retinal, which relies on IRBP for its transport, is critical for the maintenance of light sensitivity (Liou et al., 1998; Jin et al., 2009). Thus, IRBP is central to developing and maintaining the visual cycle in humans.

#### The Canonical Visual Cycle

**Figure 2:**
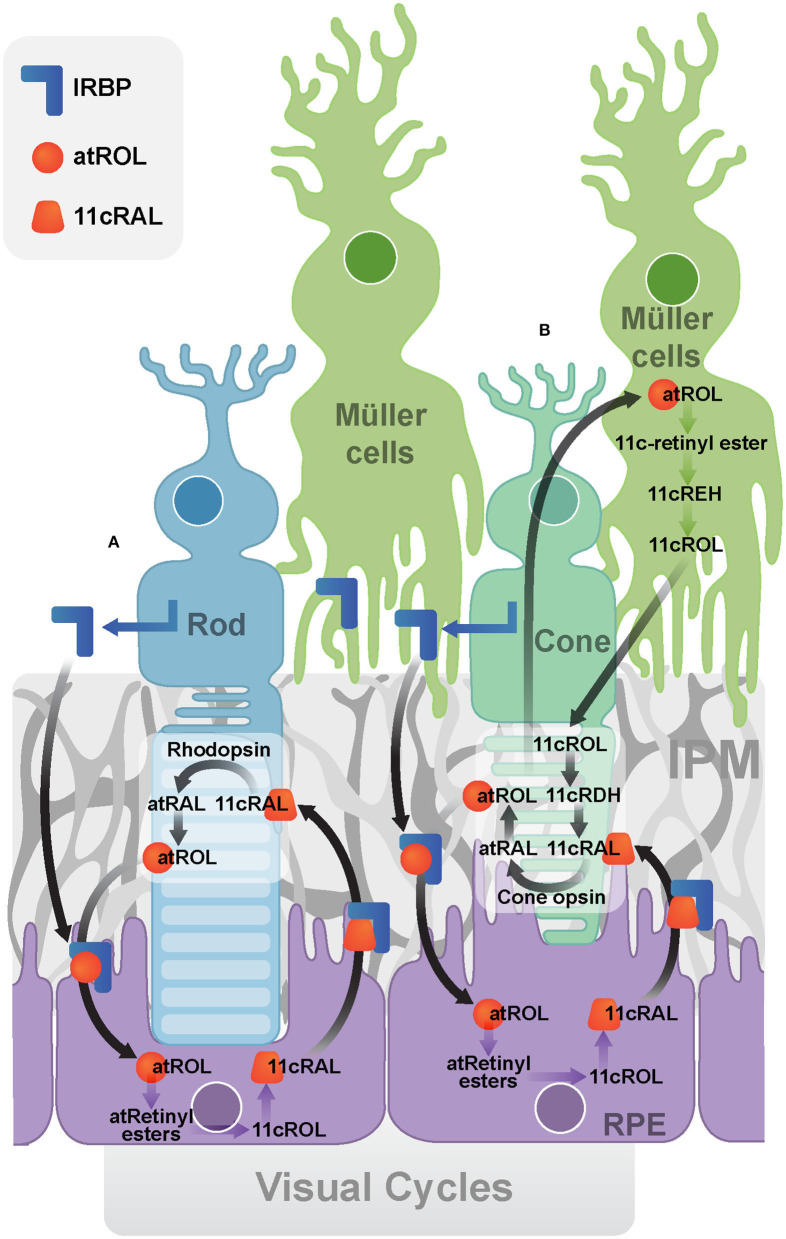
Visual Cycles. **(A)** The canonical visual cycle. IRBP facilitates the transport of retinoids (atROL and 11cRAL) between photoreceptors and the RPE. **(B)** The cone-specific visual cycle between cone photoreceptors and Müller cells. (IRBP, Interphotoreceptor retinoid-binding protein; IPM, interphotoreceptor matrix; RPE, retinal pigment epithelium; atRAL, all-*trans*-retinal; atROL, all-*trans*-retinol; 11cRAL, 11-*cis*-retinal; 11cROL, 11-*cis*-retinol; 11cRDH, 11-*cis*-retinol dehydrogenase; 11cREH, 11-*cis*-Retinyl ester hydrolase).

In the canonical visual cycle (Figure 2A), IRBP is secreted into the IPM by photoreceptors and rapidly turned over through endocytosis by photoreceptors and the RPE (Gonzalez-Fernandez, 2003). It mediates extracellular diffusion of retinoids during the operation of the retinoid cycle, transporting all-trans-retinol and 11-cis-retinal between the photoreceptors and the RPE (Gonzalez-Fernandez, 2003). A recent study suggested that IRBP may not be the only vehicle and peropsin may also transport all-trans-retinol from the neural retina to RPE (Cook et al., 2017). Human organic anion transporting polypeptide 1A2 (OATP1A2) has been recently found to be expressed at the apical membrane of RPE, where it facilitates the cellular uptake of all-trans-retinol into the RPE cells (Chan et al., 2015).

IRBP is essential for retinoid exchange in the visual cycle. Exogenously applying IRBP protein to the culture medium of rod photoreceptors reduced the level of all-trans-retinal and retinol in outer segments, preventing the formation of light-induced lipofuscin precursor (Chen et al., 2017). Supplementation of IRBP in the culture medium also facilitates the release of 11-cis-retinal from cultured fetal bovine RPE cells (Carlson and Bok, 1992). Addition of the free form IRBP to the RPE apical membrane resulted in more [3H]-11-cis-retinal release than adding cellular retinaldehyde-binding protein (CRALBP), serum retinol-binding protein (RBP), bovine serum albumin (BSA) or medium devoid of binding proteins (Carlson and Bok, 1992). Light reduced the levels of 11-cis-retinol when IRBP was absent, while it had no effect on 11-cis-retinol levels if IRBP was present. These results indicate IRBP is essential in preserving the isomeric state of retinol (Parker et al., 2011).

#### The Cone-Specific Visual Cycle

The cone-specific visual cycle (Figure 2B) refers to the exclusive ability of cone photoreceptors and Müller cells to convert all-trans-retinol to 11-cis-retinal (Wang and Kefalov, 2009; Sato and Kefalov, 2016). In contrast to the canonical visual cycle, all-trans-retinol is transported to Müller cells, facilitated by IRBP (Mata et al., 2002). IRBP was implicated by the observation that it promoted the uptake of all-trans-retinol and release of 11-cis -retinol in rat Müller cells *in vitro* (Betts-Obregon et al., 2014). Cone electroretinogram (ERG) responses in IRBP knockout mice were reduced compared to that of the control mice, although they had similar cone densities and opsin levels, indicating IRBP is essential for normal cone function or at least for the cone-specific vision cycle (Parker and Crouch, 2010). Müller cells have been found to isomerise all-trans-retinol directly to 11-cis-retinol, which then released for cone photoreceptors (Crouch et al., 1992; Das et al., 1992). Finally, the cone photoreceptor outer segments have been reported to oxidize 11-cis-retinol to 11-cis-retinal (Jones et al., 1989). Recently, further evidence has also implicated IRBP in the retinoid exchange between cones and Müller cells (Tang et al., 2013). IRBP was found to bind to the cone outer segment and Müller cell microvilli pericellular matrices (Garlipp and Gonzalez-Fernandez, 2013). This association can facilitate the delivery and uptake of its retinol ligands.

### Lipid Transport

Apart from retinoid transportation, IRBP assists the transport of various essential lipids across the IPM (Figure 1a). It has been established that IRBP contains two similar subunits with different affinities for all-trans-retinol. Long-chain fatty acids, such as Docosahexaenoic Acid (DHA), can replace all-trans-retinol from these subunits with lower affinity (Chen et al., 1993). DHA is an essential element in photoreceptor membrane biosynthesis and is thus vital for visual function in photoreceptors (Scott and Bazan, 1989; Jastrzebska et al., 2011). Post-mortem AMD retina and RPE/choroid were reported to have significantly lower levels of polyunsaturated fatty acids, such as DHA, than age-matched normal donors, suggesting the importance of IRBP to photoreceptors by transporting DHA or other fatty acids (Liu et al., 2010) (Figure 1c).

Other studies also demonstrated that other fatty acids could also compete with all-trans-retinol for binding IRBP, among which oleic acid had the highest affinity, but it was still significantly lower than that of all-trans-retinol (Semenova and Converse, 2003; Ghosh et al., 2015). A fluorometric titration experiment in which increasing the concentrations of oleic acid gradually reduced the affinity of all-trans-retinol to IRBP provided further evidence that IRBP plays a vital role in lipid transport (Ghosh et al., 2015). Competitive fluorescence and tryptophan-quenching assays also demonstrated that both oleic acid and DHA could displace all-trans-retinol from bovine IRBP, while oleic acid having a relatively higher affinity than DHA. Gas chromatography revealed that oleic acid is the most abundant fatty acid in bovine IPM, suggesting it might play an important role in maintaining the balance and transport of retinoids and fatty acids in the retina (Semenova and Converse, 2003).

### Important Roles of IRBP in IPM Integrity

The IPM, a highly organized structure between photoreceptors and the RPE, is essential for maintaining outer retinal homeostasis. Characteristic changes in IPM components occur in retinal degenerations (Ishikawa et al., 2015). For example, photoreceptor degeneration in most retinal diseases begins with the loss of the inner segments/outer segments in the IPM. However, photoreceptors may still survive under these circumstances (Goldberg et al., 2016).

IRBP accounts for ~5% of the total soluble IPM protein (Adler and Evans, 1983) and maybe crucial for the structural integrity of the IPM (Figure 1a). Although a large amount of IRBP can be removed by washout with a saline solution, other wash-resistant IRBP that co-localizes in the IPM is concentrated around cone outer segments (cone IPM-associated IRBP) (Garlipp and Gonzalez-Fernandez, 2013). Furthermore, the deduced amino acid sequence of human IRBP contains two receptors for hyaluronan-mediated motility (RHAMM)-like motifs (K321-R329 and K733-R781), suggesting a possible interaction of IRBP with the hyaluronan scaffold (Hollyfield, 1999), a component of non-covalently formed complexes with proteoglycans in the Extracellular Matrix (ECM). Thus, IRBP dysregulation is likely to affect the structural integrity of IPM, which may independently contribute to the development of many retinal diseases.

### Antioxidant Activity of IRBP

IRBP has a thiol-dependent antioxidant activity which may protect retinol from oxidation (Figure 1a). The thiol-dependent antioxidative activity of IRBP has been evaluated with an assay using myoglobin and 2,2-azinobis (3-ethylbenzothiazoline-6-sulfonic acid) (ABTS). IRBP was found to inhibit the oxidation of ABTS more actively than other free thiol groups or thiol-based reducing enzymes such as thioredoxin, vitamin E analog Trolox, and ovalbumin. Using N-ethylmaleimide (NEM) to inhibit cysteine peptidases by alkylating the active site-thiol on IRBP also suppressed the antioxidant capacity of IRBP. Thus IRBP contains a thiol-dependent antioxidant site, disruption of the putative ligand-binding site can lead to reduced antioxidant effects (Gonzalez-Fernandez et al., 2014) (Figure 1c).

All-trans-retinal causes severe cellular oxidative stress and leads to reactive oxygen species (ROS) accumulation, mitochondrial dysfunction and cell death (Maeda et al., 2009; Chen et al., 2012; Rózanowska et al., 2012). Levels of all-trans-retinal were significantly higher in the retinal explants of IRBP knockout mice than that in wild type mice after 40 min exposure to bright light. This phenomenon was significantly mitigated by adding IRBP in the culture medium (Lee et al., 2016).

## IRBP in Retinal Diseases

### IRBP in Animal Models of Retinal Disease

Extensive animal studies have shown that IRBP downregulation occurs in the early stages of many various retinal diseases. In fact, it may be a precursor to them (Zhu et al., 2015; Malechka et al., 2017). In 1989, a reduction of IRBP was reported in the early stages of retinal degeneration in a model of Abyssinian cats which carry a homozygous mutation for retinal degeneration (Narfstrom et al., 1989). A mutation in intron 50 of the centrosomal protein 290 (CEP290) gene (IVS50 + 9T > G) induced a stop codon and truncation of the mature protein in this model (Menotti-Raymond et al., 2007). IRBP-immunoreactivity was significantly reduced in the affected retina prior to the development of photoreceptor cell death (Narfstrom et al., 1989). In the same model, mRNA and protein levels of IRBP were significantly reduced as early as 4 weeks of age (Wiggert et al., 1994). Downregulation of IRBP protein was also found in a retinal degeneration 12 (rd12) mouse model with a mutation in the *RPE65* gene (Zheng et al., 2015). We have previously described this in a mouse model in which Müller cell dysfunction can be induced. A 150 kDa protein band was markedly diminished in the transgenic mice retina just 1 week after the Müller cells were disrupted, which was identified as IRBP by mass spectrometry. Photoreceptor degeneration was observed 2 weeks after the Müller cell disruption was induced in this transgenic model, suggesting that photoreceptor degeneration was secondary to Müller cell disruption (Zhu et al., 2015). It was also reported that the IRBP protein level was significantly reduced in the retinas of STZ-induced diabetic rats (Malechka et al., 2017). It has also been reported that IRBP mRNA was reduced in a light-induced retinal degeneration model in rats after animals had been exposed to intense visible light (490–580 nm green light) with an illuminance of ~1,200 lux for 24 h to induce photoreceptor degeneration (Wong et al., 2001). Overall, the early reduction of IRBP in different models of retinal degeneration suggests that IRBP may be a primary defect or an early disease marker in the retina.

IRBP dysregulation has been widely observed in different models of retinal disease. However, the consequences of IRBP downregulation on retinal or ocular health remains poorly understood. Studies on IRBP knockout models have identified an essential role of IRBP in eye growth and retinal health (Wisard et al., 2011; Markand et al., 2016). Eyes of IRBP knockout mice increased in size and weight over the wild type controls even before the mice had opened their eyelids (Wisard et al., 2011). Mice lacking IRBP display severe early and progressive photoreceptor degeneration, characterized by a reduction in both length and numbers of cone sheaths (Sato et al., 2013). IRBP knockout mice also developed profound myopia during the early stages of eye development. These eyes had longer anterior-posterior length, accompanied by a decrease in hyperopic refractive error (Markand et al., 2016). Progressive thinning of the outer nuclear layer was evident, with 20% thinning observed at postnatal day 5, and 38% thinning at day 30. Further studies, using optical coherence tomography (OCT), confirmed the previously reported retinal thinning of the outer nuclear layer in the IRBP knockout mice. Thinning of the outer nuclear layer lasted from postnatal day 15 to at least postnatal day 80 compared to wild type mice. Additionally, the slit-lamp and fundus photographs found no difference between the wild type and knockout mice (Markand et al., 2016). Another study on IRBP knockout mice revealed a loss of photoreceptor nuclei and changes in the structural integrity of retinas at postnatal day 11 and a marked loss in photic sensitivity from Electroretinography (ERG) at postnatal day 30 (Liou et al., 1998).

In summary, IRBP downregulation has been described in photoreceptors in different retinal diseases, but the precise mechanism for IRBP downregulation is still not clear. One possible explanation is that IRBP is a high molecular weight glycoprotein, the synthesis of which requires the production of large amounts of molecular building blocks and consumes a considerable amount of energy. Reduction of photoreceptor metabolism and the visual cycle may be necessary for the retina to survive under stress. Downregulation of IRBP may be beneficial for retina with short-term stress, but it might cause retinal degeneration if stress is persistent.

### IRBP and Human Retinal Diseases

It has been established that mutation, dysfunction or downregulation of IRBP can be found in several human eye diseases. For instance, a homozygous missense mutation (p.Asp1080Asn) of *IRBP* was observed in a pedigree of four patients with autosomal recessive retinitis pigmentosa (RP) (den Hollander et al., 2009). Non-sense mutations (c.1530T>A;p.Y510^*^ and c.3454G>T;p.E1152^*^) in *IRBP* were identified in four children diagnosed with retinal dystrophy and myopia (Arno et al., 2015). OCT images of these patients showed thinning of the central macula and loss of the inner segment ellipsoid band. ERG also revealed a delay and amplitude reduction in cone-specific responses (Arno et al., 2015). Li's group showed that D1080N mutation in IRBP found in patients with RP, abolished the secretion of IRBP and resulted in the formation of insoluble high molecular weight complexes via disulphide bonds. This hindered the transportation of IRBP to the Golgi and caused endoplasmic reticulum (ER) stress, which suggested another mechanism of retinal degeneration caused by IRBP mutation. A heterozygous T-C transition has been identified in autosomal recessive retinitis pigmentosa. IRBP protein was at a significantly lower level in aqueous humor of primary congenital glaucoma patients (Li et al., 2013). Reduced IRBP mRNA and protein expression were observed in the retinas from diabetic donors when compared with those from non-diabetic donors (Garcia-Ramirez et al., 2009). Analysis of vitreous fluid obtained from clinics revealed that IRBP levels were reduced in the early stages of diabetic retinopathy (Garcia-Ramirez et al., 2009). Vitreous IRBP concentration declined gradually with the increasing severity of diabetic retinopathy in eyes with established retinopathy. IRBP protein levels both in the retina and vitreous of eyes with non-proliferative diabetic retinopathy were higher than those with proliferative diabetic retinopathy (Yokomizo et al., 2019).

Downregulation of IRBP also caused the accumulation of the retinal 'waste product' lipofuscin, which may increase the risk of oxidative damage to the RPE and photoreceptors (Radu et al., 2003) (Figure 1c). Lipofuscin is also responsible for retinal autofluorescence in retinal diseases (Birnbach et al., 1994; Marmorstein et al., 2002; Radu et al., 2003). Studies on cryosections of human retinas with AMD have revealed that lipofuscin in the RPE was strongly autofluorescent (Marmorstein et al., 2002). Stargardt's disease is characterized by hyper-autofluorescence and loss of macular photoreceptors which have been correlated with clinical progression of the disease (Birnbach et al., 1994).

We have discussed IRBP dysregulation as an early disease indicator, playing a role in the early stages of retinal pathology. Further studies are required to elucidate all of the mechanisms by which IRBP dysfunction may contribute to retinal disease pathogenesis.

### Autoantibodies to IRBP

The retina is an 'immune-privileged' site, which means it may tolerate the introduction of antigens without eliciting an inflammatory immune response (Benhar et al., 2012). This presumably protects the retina from potentially blinding processes such as fibrosis that may result from inflammation. The blood-retinal barrier (BRB) is the interface between systemic circulation and the retina, which is critical for the maintenance of this immune-privilege (Benhar et al., 2012). Breakdown of the BRB, which occurs in many retinal diseases, exposes retinal antigens to the immune system, eliciting an inflammatory response leading to tissue damage and vision loss (Chen et al., 2019).

IRBP peptides can induce experimental autoimmune uveitis, which is a well-estblished model of uveitis (Caspi et al., 1990), as well as the effects of the adaptive immune response in the eye (Agarwal et al., 2012; Kyger et al., 2013). Anti-IRBP autoantibodies have been found in patients with uveitis, RP, Coats disease, AMD and Macular Telangiectasia Type 2 (MacTel) (Solomon et al., 1999; Morohoshi et al., 2012; Kyger et al., 2013; Zhu et al., 2013; Gibbs et al., 2017). A study investigated the autoimmune condition of a girl with a rare triad of RP, Coats disease and uveitis, and found that her peripheral lymphocytes had a specific anamnestic response to IRBP (Solomon et al., 1999), indicating that autoimmunity toward IRBP might play a role in the degeneration of photoreceptors. The IRBP autoantibody was detected in 28% (5 out of 18) of patients with non-infectious uveitis (Gibbs et al., 2017), in 33% (6 out of 18) of patients with AMD and in 24% (11 out of 45) of those with MacTel (Zhu et al., 2013). The detection of IRBP autoantibodies in these patients suggested that such diseases may share some common etiological or pathogenic mechanisms (Zhu et al., 2013). Significant downregulation of IRBP protein expression was also detected in the retina of mice with induced Müller cell dysfunction, which is a model of primary Müller cell loss that has been implicated in the pathogenesis of MacTel (Zhu et al., 2015). Whether these autoantibodies actually cause the reduction of IRBP or whether they are an epiphenomenon is still uncertain, but it appears certain that loss of IRBP is closely related to many retinal diseases.

## The Potential Applications of IRBP in Treatment for Retinal Diseases

IRBP could be a potential novel target in treating retinal diseases, considering its essential role in the maintenance of the visual cycle and other physiological functions in the IPM (Gonzalez-Fernandez et al., 2014; Ghosh et al., 2015; Chen et al., 2017). The upregulation of IRBP prevented photoreceptor degeneration in diabetic mice and rats through the regulation of VEGF (Yokomizo et al., 2019). This data indicates that IRBP may also be beneficial in other diseases characterized by photoreceptor degeneration and VEGF dysregulation, such as AMD. Downregulation or dysregulation of IRBP could disrupt the visual cycle which leads to the accumulation of the all-trans-retinal, one component of retinal “waste product” lipofuscin. The effect of IRBP on preventing lipofuscin accumulation could be central for AMD-like diseases (Radu et al., 2003) (Figure 1c). Direct upregulation of IRBP through inducing the whole IRBP gene (9.5 k base pairs) warrants further investigation, but the difficulty is that the genetic constructs required would exceed the packing capacity of traditional adenovirus carriers for gene therapy. The insert size could be even longer after adding additional cell-specific promoters such as promoter fragments of cone transducin α (TαC), rhodopsin kinase (GRK1) or cone arrestin (CAR) gene (Kennedy et al., 2007; McDougald et al., 2019) for target-specific expression in photoreceptors. Here, we discuss three alternative ways to upregulate IRBP expression (small molecules, microRNAs, and CRISPRa technique), which we anticipate will protect photoreceptors from degeneration.

### Small Molecules

Chemical communication mediated by molecular signaling coordinates cell behavior (Buddingh et al., 2020). Many natural or synthetic chemical compounds which regulate different metabolic or signaling pathways have great therapeutic potential. Small molecules that have the antioxidant, anti-inflammatory and anti-excitotoxic capacity in the central nervous system can be administered to protect photoreceptors (Zhang et al., 2019). For example, simvastatin is a cholesterol-lowering drug and was recently reported to protect Y79 retinoblastoma cells which have many characteristics of photoreceptors, through upregulating IRBP and its transcription factor CRX (Zhang et al., 2019). Some other small molecules acting on the cardiovascular system may also have a neuroprotective effect on photoreceptors (Zhao et al., 2016). Tetramethylpyrazine, which has been widely used in treating cardiovascular diseases for over 40 years, was found to attenuate all-trans-retinal -induced cytotoxicity in the differentiated Y79 cells via suppressing oxidative and nitrosative stress, apoptosis and leukostasis (Zhao et al., 2016). A further study found that the neuroprotective effect of tetramethylpyrazine against all-trans-retinal toxicity is also mediated through upregulating IRBP expression (Wang et al., 2017). Therefore, some small molecules have the potential to protect photoreceptors from stress by increasing IRBP expression.

Small molecules, however, have several shortcomings. Firstly, the downstream signaling cascade reactions and information encoding may vary broadly with different concentrations of signaling molecules (Purves and Fitzpatrick, 2001). Thus, the optimal dosage must be precisely controlled. Also, the enormous quantity of small molecules makes it difficult to find the desired molecule accurately. A luciferase reporter system may overcome this drawback (Xie et al., 2016). The luciferase reporter system can be specifically designed to screen small molecules that upregulate IRBP expression. By inserting the promoter region of human IRBP gene into the luciferase reporter plasmid, small molecules that specifically bind to this promoter region can be identified from the luciferase reporter activity (Miraglia et al., 2011). However, it is difficult to disentangle the downstream signaling through which the candidate small molecules regulate the expression of IRBP. Some molecules acting on Müller cells may also modulate IRBP expression via complex glia-neuron interactions (Vecino et al., 2016). Low specificity, low efficiency and the short-acting time of small molecules also pose a formidable challenge to their successful application (Maxim et al., 2014). A sustained-release formulation is highly desirable in this case. Nanoparticles introduced in the vitreous may sustain the delivery of the encapsulated agents for longer durations (Kompella et al., 2003; Riley and Vermerris, 2017). For example, Anti-VEGF aptamer EYE001 (tested in humans for efficacy) entrapped in Poly lactic-co-glycolic acid (PLGA) microspheres were found to deliver EYE001 in a sustained manner with retained activity *in vitro* and *ex vivo* (Carrasquillo et al., 2003). Lipid nanoparticle approaches with specific and sustained delivery systems are expected to gain more attention in the future. Several other studies have also demonstrated the successful application of nanoparticles in the liver with approval by the US Food and Drug Administration (FDA) (Coelho et al., 2013; Rizk and Tuzmen, 2017).

Therefore, the successful clinical application of synthetic and natural compounds may provide advantages but will present challenges.

### MicroRNAs

A microRNA is a small non-coding RNA that can regulate gene expression of complementary mRNAs by binding to the 3′ untranslated region (3′ UTR) (Ambros, 2004; Bartel, 2018). More than 60% of protein-encoding genes are controlled by microRNAs (Friedman et al., 2009). It is recognized that microRNAs play an important role at the post-transcriptional level through degradation and translational repression of their target mRNAs (He and Hannon, 2004). Since the first study in 1993 discovering these post-transcriptional RNA-RNA interactions, microRNAs have attracted lots of attention due to their powerful post-transcriptional role, small size (21 nt), ease of transfection and ability for a single miRNA to regulate whole gene pathways (Lee et al., 1993; Wightman et al., 1993; Filipowicz et al., 2008). 320 and 340 different kinds of microRNAs have been found in mouse retina and RPE/choroid, respectively (Soundara Pandi et al., 2013). Despite miRNA being suggested as being involved in retinal degenerations, no studies have looked at the role of miRNA regulation on IRBP.

Computational analysis using target scan (http://www.targetscan.org/vert_72/) (Agarwal et al., 2015), which used to predict related microRNAs, showed no validated sequences for IRBP gene, but several predicted sequences. Predicted IRBP-related microRNAs have been studied in previous retinal studies, as shown in Table 1. Among which, miR-140-3p, miR-210-5p and miR-190b-5p may be of interest in manipulating the expression of IRBP. miR-140-3p participates in RPE cell survival and apoptosis. Loss of circRNA_0084043 depressed high glucose (HG)-induced apoptosis in ARPE-19 cells by upregulating miR-140-3p (Li et al., 2020). Given the essential role inflammation plays in retinal degenerations, miRNA that simultaneously maintains normal retinal function and influence inflammatory processes are of key therapeutic interest.

**Table 1:** Correlation of predicted microRNA in regulating IRBP and retinal disease.

**Predicted miRNA**	**Reported miRNA**	**Regulation**	**Site**	**Disease or model**	**References**	**Others**
hsa-miR-152-5p	hsa-miR-152	Down	Human vitreous	AMD vs. Con	Ménard et al., 2016	
hsa-miR-22-3p	hsa-miR-22	Down	Human retina	AMD vs. Con	Lukiw et al., 2012	
hsa-miR-146b-3p	Same	Down	Human vitreous	Diabetic vs. Con	Fulzele et al., 2015	
hsa-miR-3121-5p	hsa-miR-3121	Up	Human serum	dry AMD vs. Con	Szemraj et al., 2015	
hsa-miR-1306-5p	Same	Up	Human plasma	Glaucoma & XFS vs. Con	Hindle et al., 2019	
hsa-miR-3173-5p	hsa-miR-3173	Up	Human aqueous humor	Glaucoma & XFS vs. Con	Hindle et al., 2019	
hsa-miR-4448	Same	Up	Human aqueous humor	Glaucoma & XFS vs. Con	Hindle et al., 2019	
hsa-miR-152-5p	hsa-miR-152	Down	hREC	HG condition vs. Con	Haque et al., 2015	*In vitro*
hsa-miR-185-3p	Same	Down	Rabbit retina	Newborn vs. Adult	Robert et al., 2010	Rabbit
hsa-miR-18a-3p	Same	Down	Human aqueous humor	POAG vs. Cataract	Liu et al., 2018	
hsa-miR-410-3p	Same	Up	Human aqueous humor	POAG vs. Cataract	Liu et al., 2018	
hsa-miR-4433b-3p	Same	Up	Human aqueous humor	POAG vs. cataract	Liu et al., 2018	
hsa-miR-487a-5p	Same	Up	Human aqueous humor	POAG vs. Cataract	Liu et al., 2018	
hsa-miR-501-3p	Same	Up	Human aqueous humor	POAG vs. Cataract	Liu et al., 2018	
hsa-miR-760	Same	Up	Human aqueous humor	POAG vs. Cataract	Liu et al., 2018	
hsa-miR-874-3p	Same	Down	Human aqueous humor	POAG vs. Cataract	Liu et al., 2018	
hsa-miR-3149	Same	Up	Human serum	POAG vs. Con (cataract included)	Liu et al., 2019	
hsa-miR-18a-3p	hsa-miR-18a	Up	Human retina	RB vs. Con	Martin et al., 2013	
hsa-miR-22-3p	hsa-miR-22	Down	Human retina	RB vs. Con	Martin et al., 2013	
hsa-miR-504-3p	hsa-miR-504	Down	Human retina	RB vs. Con	Martin et al., 2013	
hsa-miR-874-3p	hsa-miR-874	Down	Human retina	RB vs. Con	Martin et al., 2013	
hsa-miR-214-3p	Same	Down	Human plasma	ROP vs. Con	Metin et al., 2018	
hsa-miR-223-5p	Same	Up	Human vitreous	severe PVR vs. mild PVR	Toro et al., 2020	
hsa-miR-1909-5p	Same	Up	ARPE-19 cell	TGFβ2 induced EMT vs. Con	Chen et al., 2014	*In vitro*
hsa-miR-223-5p	Same	Up	ARPE-19 cell	TGFβ2 induced EMT vs. Con	Chen et al., 2014	*In vitro*
hsa-miR-146b-3p	hsa-miR-146b-5p	Down	Human plasma	wet AMD vs. Con	Ertekin et al., 2014	
hsa-miR-324-3p	Same	Up	Human plasma	wet AMD vs. Con	Ertekin et al., 2014	Express only in patient group
hsa-miR-410-3p	hsa-miR-410	Down	Human plasma	wet AMD vs. Con	Ertekin et al., 2014	
hsa-miR-574-5p	hsa-miR-574-3p	Down	Human plasma	wet AMD vs. Con	Ertekin et al., 2014	

*hREC, human retinal endothelial cells; XFS, exfoliation syndrome; Con, control; AMD, age-related macular degeneration; HG, high glucose; RB, retinoblastoma; POAG, primary open-angle glaucoma; EMT, epithelial-mesenchymal transition; ROP, retinopathy of Prematurity; PVR, proliferative vitreoretinopathy*.

Once such miRNA miR-146a downregulates various genes involved in normal retinal function and homeostasis, including IL-6, IL-8 (Chen et al., 2010; Li et al., 2010) and is also predicted to regulate IRBP. The exploitation of microRNAs involved in modulating IRBP may be of clinical significance.

### CRISPRa Gene Therapy

Since the development of gene-editing techniques such as zinc-finger nucleases (ZFNs) and transcription activator-like effector nucleases (TALENs), the third generation, clustered regularly interspaced short palindromic repeats (CRISPR) technique has recently risen (Doudna and Charpentier, 2014). This technique has made gene therapy easier and more specific for targeting a gene of interest, like IRBP. The recent development of CRISPR interference (CRISPRi) and CRISPR activation (CRISPRa) system that fuses dead nuclease Cas9 (dCas9) to a transcriptional complex, enables inhibiting or activating the transcription of target genes rather than cleaving them (Gilbert et al., 2014). CRISPRa technology also can activate multiple genes simultaneously (McCarty et al., 2020). Multiplex modulation through CRISPRa enables more precise and efficient gene editing, as many human diseases result from mutations in multiple genes (Zlotogora, 2007). Human IRBP is ~9.5 kb in length, which exceeds the standard packing capacity of a virus. This is a significant hurdle to develop gene therapies that target IRBP. We may benefit from boosting the transcription of IRBP through specifically designed guide RNA in the CRISPRa system. Gene therapies using CRISPR technology have already been launched in clinical trials. A patient with a hereditary blindness disorder has become the first to receive a CRISPR/Cas9 gene therapy administered directly into their body recently (Ledford, 2020). Thus, the success of IRBP upregulation using this advanced technology has translational applications.

## Conclusion and Future Directions

IRBP is required to maintain the normal functions of the retina, and its downregulation is a common phenomenon at the early stages of photoreceptor degeneration. Although it may be an initial defensive response to retinal stress, the suppression of IRBP is harmful to the health of the photoreceptors in the long term (Figure 1c). The close relationship of IRBP downregulation with early symptoms and retinal disease severity forms the basis of its clinical applications as an early diagnostic marker and therapeutic target for many retinal diseases (Garcia-Ramirez et al., 2009; Zhu et al., 2015; Yokomizo et al., 2019). It is expected that restoring the expression of IRBP may slow the degeneration of photoreceptors. Future research involving techniques like CRISPRa-based gene therapy will allow for further exploration of the clinical potential of treating retinal diseases with IRBP-targeted gene therapy.

## Author Contributions

TZ and LZ conceived the perspective of the work. SZ, TZ, YC, LH, and MP searched and summarized the literature. SZ and TZ drafted the manuscript. TZ, RA-B, LZ, and RN designed the figure. MG, NF, RN, FZ, and MM critically revised the article. All authors commented on and contributed to the final manuscript.

## Conflict of Interest

The authors declare that the research was conducted in the absence of any commercial or financial relationships that could be construed as a potential conflict of interest.
